# Postoperative opioids administered to inpatients with major or orthopaedic surgery: A retrospective cohort study using data from hospital electronic prescribing systems

**DOI:** 10.1371/journal.pone.0305531

**Published:** 2024-06-25

**Authors:** Yun-Ting Huang, William G. Dixon, Terence W. O’Neill, Meghna Jani

**Affiliations:** 1 Centre for Epidemiology Versus Arthritis, Centre for Musculoskeletal Research, University of Manchester, Manchester, United Kingdom; 2 NIHR Manchester Biomedical Research Centre, Manchester University NHS Foundation Trust, Manchester Academic Health Science Centre, Manchester, United Kingdom; 3 Department of Rheumatology, Salford Royal Hospital, Northern Care Alliance, Salford, United Kingdom; IRCCS: IRCCS Ospedale San Raffaele, ITALY

## Abstract

**Background:**

Opioids administered in hospital during the immediate postoperative period are likely to influence post-surgical outcomes, but inpatient prescribing during the admission is challenging to access. Modified-release(MR) preparations have been especially associated with harm, whilst certain populations such as the elderly or those with renal impairment may be vulnerable to complications. This study aimed to assess postoperative opioid utilisation patterns during hospital stay for people admitted for major/orthopaedic surgery.

**Methods:**

Patients admitted to a teaching hospital in the North-West of England between 2010–2021 for major/orthopaedic surgery with an admission for ≥1 day were included. We examined opioid administrations in the first seven days post-surgery in hospital, and “first 48 hours” were defined as the initial period. Proportions of MR opioids, initial immediate-release(IR) oxycodone and initial morphine milligram equivalents (MME)/day were calculated and summarised by calendar year. We also assessed the proportion of patients prescribed an opioid at discharge.

**Results:**

Among patients admitted for major/orthopaedic surgery, 71.1% of patients administered opioids during their hospitalisation. In total 50,496 patients with 60,167 hospital admissions were evaluated. Between 2010–2017 MR opioids increased from 8.7% to 16.1% and dropped to 11.6% in 2021. Initial use of oxycodone IR among younger patients (≤70 years) rose from 8.3% to 25.5% (2010–2017) and dropped to 17.2% in 2021. The proportion of patients on ≥50MME/day ranged from 13% (2021) to 22.9% (2010). Of the patients administered an opioid in hospital, 26,920 (53.3%) patients were discharged on an opioid.

**Conclusions:**

In patients hospitalised with major/orthopaedic surgery, 4 in 6 patients were administered an opioid. We observed a high frequency of administered MR opioids in adult patients and initial oxycodone IR in the ≤70 age group. Patients prescribed with ≥50MME/day ranged between 13–22.9%. This is the first published study evaluating UK inpatient opioid use, which highlights opportunities for improving safer prescribing in line with latest recommendations.

## Introduction

Opioids are an important treatment option in the management of postoperative pain. Poorly controlled postoperative pain may result in increased morbidity, poor quality of life and impaired functional recovery [1]. Research shows up to 10% of opioid-naïve patients who take opioids in the postoperative period transition to persistent opioid use [1], an outcome that is associated with poor health outcomes such as opioid dependence, abuse and harms [2]. Postoperative opioid prescribing therefore has attracted public attention and concern because it can lead to persistent opioid use, which has been a potential contributor to the opioid crisis in North America and other Western countries [3]. However, most evidence for postoperative opioid prescribing has been extrapolated from North America and it has been acknowledged on a national level that it has not been possible to quantify this problem in the UK [4].

There has been emerging evidence suggesting specific opioids and/or formulations are less preferable for postoperative pain management. Modified-release (MR) opioids have been shown to be associated with an increased risk of persistent opioid use, opioid-related harms, and longer hospital stay [5–7]. Their slow onset and offset also make dose titration less flexible when patients recover from surgery and need to titrate down opioids rapidly and safely [5]. Therefore immediate-release (IR) opioids are the preferred management choice in postoperative pain, to reduce the risk of respiratory depression and persistent use [4]. Specifically, fentanyl transdermal patches have been deemed by the Medicines and Healthcare Products Regulatory Agency (MHRA) to be contraindicated in opioid-naive patients in the UK in 2020, and therefore are not recommended as regular treatments for post-surgical pain [8]. Oral oxycodone, a drug available in either IR or MR format, has emerged as a drug with higher drug abuse liability than other opioids in recent years, and thus becomes less favourable [9,10]. Marketing of oxycodone MR (i.e., OxyContin) is widely acknowledged to be a factor contributing to the opioid epidemic in the US [11]. Moreover, oxycodone in IR forms is not recommended as a first-line opioid as it is a schedule 2 drug (i.e. with some medically acceptable uses, but with high potential for abuse and/or addiction), as per the latest best practice guidelines published by the Royal College of Anaesthetists (RCoA) [4]. Patients aged >70 years or with renal impairment, who have changes in body composition and may metabolise drugs differently, may be more vulnerable to opioid-related harms. It is therefore acknowledged such patients may have different opioids prescribed, however national guidance doesn’t specify which ones.

Hospital inpatient prescribing which includes the immediate post-operative period is more difficult to access for research due to the absence of well curated research datasets unlike UK primary care or US administrative data. Additionally, most studies focusing on primary care opioid prescribing do not provide information on whether the patient was administered the medication in hospital, leading to some exposure misclassification. Hospital data provides opportunity to assess administered use of such medications, especially when a considerable proportion of opioids may be prescribed on an as required basis [12].

To date, most of the literature defines postoperative opioid use as having an opioid prescription at or after discharge into the community. The period after hospital discharge varies greatly from seven days to six months [13–15]. Reported findings have been mixed, most focussing on postoperative opioid prescribing in community/ at discharge in North American jurisdictions using administrative datasets [16–18]. Postoperative opioid use data in the UK are scarce, for example, one service evaluation in a cohort of 499 patients that assessed opioids prescribed at discharge [19] and one study linking primary and secondary care data to evaluate persistent postoperative opioid use post-discharge [20]. Such evaluations, however, miss opioid prescribing in the immediate postoperative period (i.e., in hospital) which may influence subsequent outcomes such as persistent opioid use. In order to design and target future interventions, accessing high-quality hospital data to understand current practices in opioid prescribing between surgery and discharge is imperative.

The aim of this study was to utilise administered drug data from inpatient electronic prescribing (ePrescribing) systems to examine postoperative opioid patterns during the hospital stay for people admitted to a large teaching hospital due to major or orthopaedic surgery between 2010 and 2021. The objectives were to assess the 1) proportion of patients administered opioids following surgery 2) opioid utilisation patterns with focus on opioid mono- or combination therapy, MR opioids, initial use of oxycodone IR and initial morphine milligram equivalents (MME)/day, 3) proportion of patients prescribed any opioid at discharge. A subgroup of patients aged >70 years or with renal impairment was analysed separately.

## Materials and methods

### Study design and population

This retrospective cohort study included patients who were admitted to a teaching hospital in the North West of England between 1/1/2010 and 31/08/2021 due to major or orthopaedic surgery. Major surgery included surgery to the organs of the head, chest and abdomen, such as removal of a brain tumour, open-heart surgery, organ transplant or appendicectomy. Examples of orthopaedic surgery were knee or hip replacement surgery and ligament reconstruction. We identified surgery types using Operating Procedure Codes Supplement-4 (OPCS-4) codes, a statistical classification for clinical coding of hospital interventions and procedures undertaken by the NHS [21]. The codes were developed by clinical academic members of the study team, and we included patients who had the relevant OPCS-4 codes recorded in the primary procedure as their main reason for admissions. A patient might have more than one hospital admission in the study period and each hospital admission was treated as a unique episode. Only those patients who stayed in the hospital for at least one day (i.e., discharged on the next day) and had been administered opioids after surgery were included for the main analysis; those who had been on opioids only between hospital admission and one day before surgery (i.e., no opioids from surgery day onwards) were excluded. Patients on opioids given by intrathecal, epidural, or intranasal routes only were also excluded.

### Patient characteristics

Patient characteristics including age, gender, ethnicity and the Index of Multiple Deprivation (IMD) were recorded for each episode for each individual. IMD scores were derived from postcodes using the English IMD 2019 and further categorised into quintiles [22]. Only patients with a postcode in England had IMD data as IMD scores were not comparable between England and Wales/ Scotland/ Northern Ireland. Those without IMD information were coded as a separate category and included in the analyses. Co-existing comorbidities were recorded using the International Classification of Diseases 10th Revision (ICD10) codes in the discharge summary and assessed using the Charlson Comorbidity Index (CCI) [23].

### Opioid exposure and utilisation patterns

Administration of opioids was assessed at a patient level, based on ePrescribing records from between the date of surgery up to the point of discharge. All opioids were extracted, the most common included buprenorphine, codeine, diamorphine, dihydrocodeine, fentanyl, hydromorphone, meptazinol, morphine, oxycodone, pethidine, and tramadol, reflecting the frequently prescribed opioids used in the UK [24]. Initial opioid use was defined as opioids administered within “first 48 hours” post-surgery. Discharge medications were also extracted from inpatient ePrescribing systems. The immediate postoperative period was classed as the first seven days following surgery. In those patients, we evaluated:

1. Patients who had opioids administered following surgery
2. Opioid utilisation patterns including:
  - Different opioid regimens defined as monotherapy or combination therapy
  - MR opioids, defined as use at least once, including prolonged-released oral forms and transdermal forms, and fentanyl patches, by calendar year
  - Initial use of oxycodone IR, by calendar year
  - Initial MME/day, by calendar year
3. Patients who were prescribed any opioid at discharge

For patients aged >70 years or with renal impairment, initial opioid use was assessed separately by looking at the trends of MME/day between 2010–2021 and the proportion of IR/ MR opioid type (e.g., codeine IR).

Some administrations, such as patient-controlled analgesia (PCA) and injections specifically during immediate recovery only, were excluded from MME calculations. This was because PCA administration lacked accurate dose information and recovery-only injections were not given for post-surgical pain control. MME/day was determined by converting each opioid medication’s daily dosage using the equivalent analgesic ratio released by the Centres for Disease Control and Prevention (CDC) [25]. If patients were on more than one opioid, the MME/day for each administered drug was added together, to derive the total value per day. For transdermal and infusion formulations, strength per hour and the duration of administration were considered in the dose calculation to avoid underestimation of MME/day. Mean value of MME/day for the first 48 hours after surgery was calculated to assess the initial dosage. MME/day was classified into three categories: <50, 50–119 and ≥120 as defined previously [24]. All data analyses were performed using R (version 4.1.3). The study received ethics approvals from both the Health Research Authority (Reference 21/LO/0556) and University Research Ethics Committee (Reference: 2021-12958-20789). Data were accessed for research purposes on the 1^st^ of November 2021. The researchers did not have access to identifiable information of the participants in the study as all data were pseudonymised as per the approvals of the study. Informed consent was not required in line with the ethics approvals of the study as no identifiable patient data was available to the research team.

## Results

### Baseline characteristics

Among patients admitted to the hospital for major or orthopaedic procedures, 71.1% of the patients (i.e., 57.0% of the admissions) had opioids administered during their hospitalisation. The cohort derivation flowchart is shown in S1 Fig. This study included 60,167 unique hospital admissions from 50,496 unique patients administered an opioid between 2010–2021, with 23,487 (46.5%) having major surgery and 27,009 (53.5%) undergoing orthopaedic surgery.

The mean age of all patients was 55.5 years (standard deviation (SD) = 18.1 years) (Table 1). Patients undergoing major surgery had a longer hospitalisation (median = 5, interquartile range (IQR):2–12) compared to those with orthopaedic surgery (median = 3, IQR:1–10). Around half of the patients were females and the majority (93.1%) were white. Over a third (37.7%) of the cohort were in the most deprived quintile according to the English IMD 2019 classification [22], and around 1% had missing IMD. Patients with major surgery had a higher proportion of baseline cerebrovascular disease compared with those who underwent orthopaedic surgery (8.3% vs. 2.2%) and cancer (22.8% vs. 3.6%), and a lower proportion of baseline rheumatic disease (1.5% vs. 3%). The most frequent surgical procedures are presented in S1 Table. Primary decompression operations on lumbar spine (6.9%) were the most common, followed by primary excision of cervical intervertebral disc (3.0%), excision of gall bladder (2.8%) and drainage of subdural space (2.0%). The high numbers of spine and neurosurgical procedures reflect the fact that the teaching hospital has a large Complex Spine Surgery Department and a specialist neurosurgical unit.

**Table 1:** Baseline characteristics among 50,496 patients between 2010–2021.

	Major surgery (N = 23,487)	Orthopaedic surgery (N = 27,009)	Total (N = 50,496)
Age (in years), mean (SD)	54.0 (17.3)	56.8 (18.8)	55.5 (18.1)
Hospital stay (in days), median (IQR)*	5 (2–12)	3 (1–10)	4 (2–11)
Female, N (%)	12164 (51.8%)	13623 (50.4%)	25787 (51.1%)
Ethnicity, N (%)			
White	21703 (92.4%)	25303 (93.7%)	47006 (93.1%)
Non-white or mixed	1784 (7.6%)	1706 (6.3%)	3490 (6.9%)
IMD quintiles^†^, N (%)			
1 (Most deprived)	8827 (37.6%)	10224 (37.9%)	19051 (37.7%)
2	4863 (20.7%)	5662 (21.0%)	10525 (20.8%)
3	3363 (14.3%)	3890 (14.4%)	7253 (14.4%)
4	3221 (13.7%)	3863 (14.3%)	7084 (14.0%)
5 (Least deprived)	2874 (12.2%)	3202 (11.9%)	6076 (12.0%)
Comorbidity, N (%)			
Myocardial infarction	651 (2.8%)	834 (3.1%)	1485 (2.9%)
Congestive heart failure	479 (2.0%)	803 (3.0%)	1282 (2.5%)
Peripheral vascular disease	445 (1.9%)	421 (1.6%)	866 (1.7%)
Cerebrovascular disease	1942 (8.3%)	592 (2.2%)	2534 (5.0%)
Dementia	167 (0.7%)	946 (3.5%)	1113 (2.2%)
Chronic pulmonary disease	3249 (13.8%)	3629 (13.4%)	6878 (13.6%)
Rheumatic disease	345 (1.5%)	808 (3.0%)	1153 (2.3%)
Peptic ulcer disease	192 (0.8%)	29 (0.1%)	221 (0.4%)
Mild liver disease	667 (2.8%)	412 (1.5%)	1079 (2.1%)
Diabetes without complications	2434 (10.4%)	2463 (9.1%)	4897 (9.7%)
Diabetes with complications	203 (0.9%)	330 (1.2%)	533 (1.1%)
Hemiplegia or paraplegia	611 (2.6%)	977 (3.6%)	1588 (3.1%)
Renal disease	1042 (4.4%)	1306 (4.8%)	2348 (4.6%)
Cancer (any malignancy)	5354 (22.8%)	985 (3.6%)	6339 (12.6%)
Moderate or severe liver disease	59 (0.3%)	49 (0.2%)	108 (0.2%)
Metastatic solid tumour	1325 (5.6%)	679 (2.5%)	2004 (4.0%)

Abbreviations: SD = standard deviation; IQR = interquartile range; IMD = Index of Multiple Deprivation.

One patient may have more than one hospital admission during the study period. We reported baseline characteristics based on the first admission of one patient.

* IQR = interquartile range.

^†^ IMD has 1% (n = 507) missingness in total due to no comparable information on postcodes from Wales, Scotland and Northern Ireland.

### Opioid regimens

**Fig 1:**
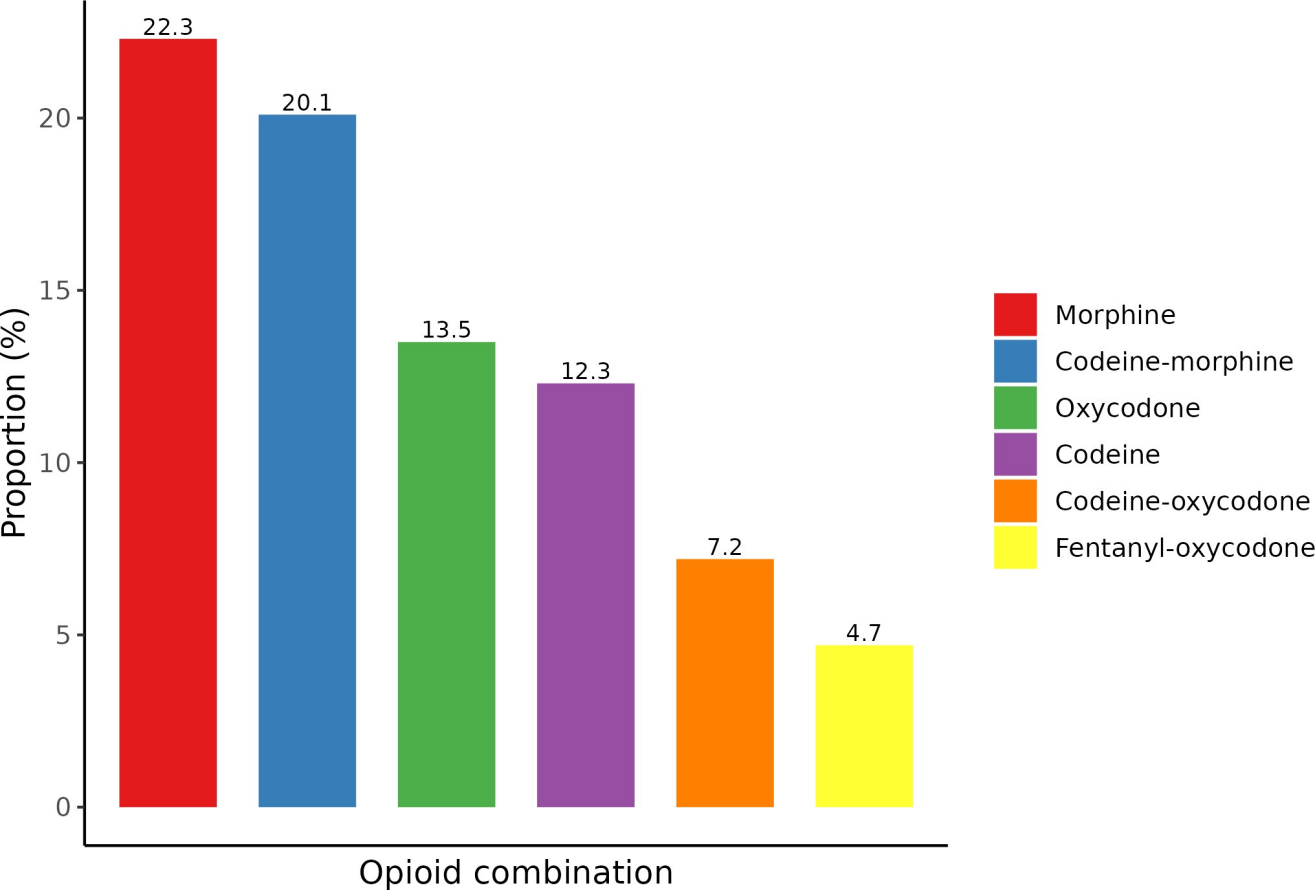
Most frequent (top six) inpatient opioid regimens within one-week post-surgery. The sum of the proportions in this figure is less than 100% because less common regimens are not shown (see S2 Fig for alternative regimens). A total of 59,062 admissions with opioids administered in the first seven days between 2010–2021 were included.

Morphine, codeine and oxycodone were the most commonly administered opioid treatments for post-surgery inpatients (Fig 1). Morphine monotherapy was the most common regimen (22.3%), followed by morphine and codeine combination (20.1%), oxycodone monotherapy (13.5%), codeine monotherapy (12.3%), codeine plus oxycodone combination (7.2%) and fentanyl plus oxycodone combination (4.7%). Less common opioid regimens, including opioid combinations, are presented in S2 Fig (1–3% of administrations).

### Trends for MR opioids, initial oxycodone IR, and initial MME/day

**Fig 2:**
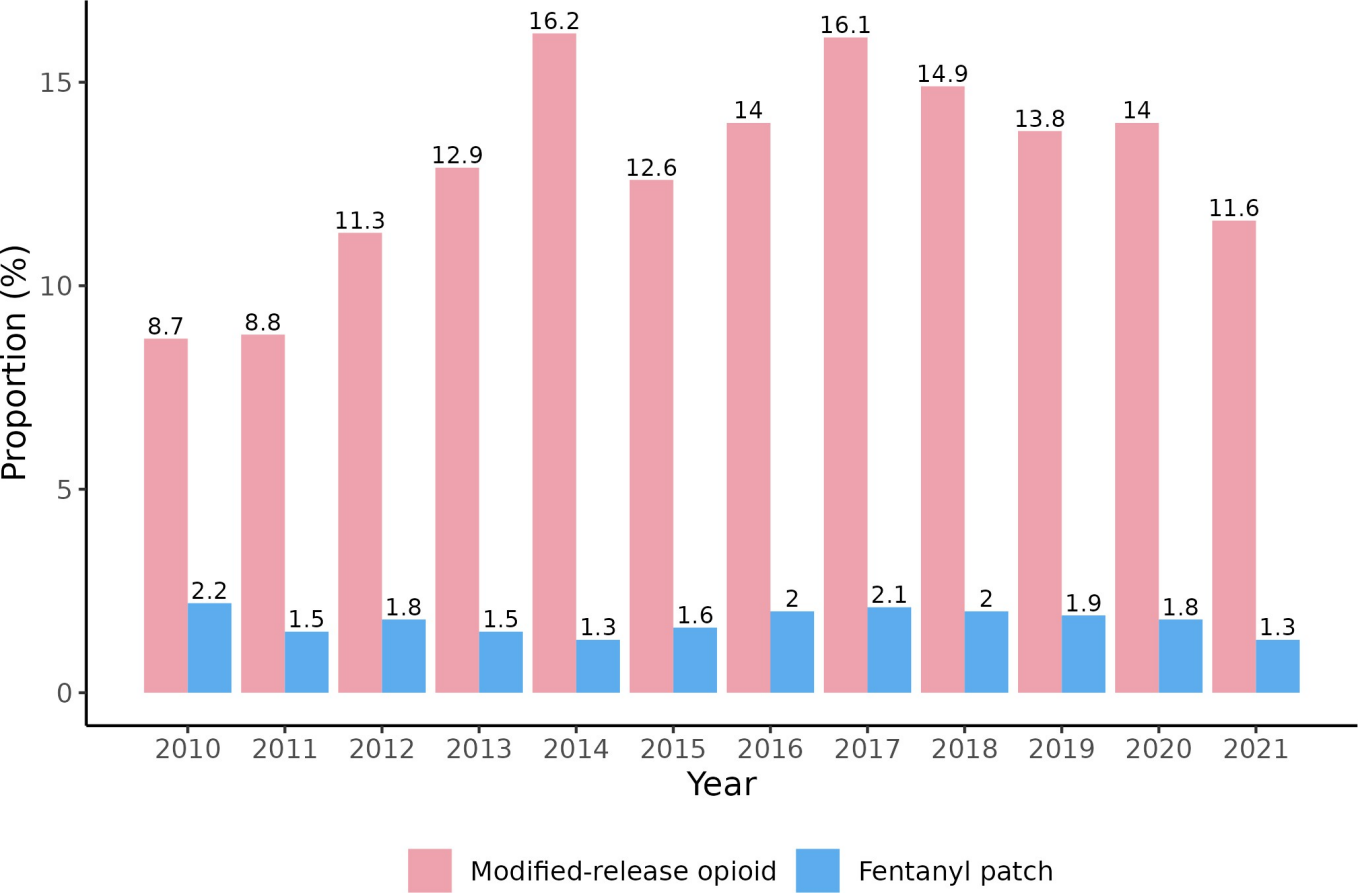
Trends of modified-release opioids and fentanyl patches within one-week post-surgery between 2010–2021. A total of 59,062 admissions with opioids administered in the first seven days between 2010–2021 were included. Each year had different numbers of admissions as the denominator, ranging from 2,064 to 6,329.

**Fig 3:**
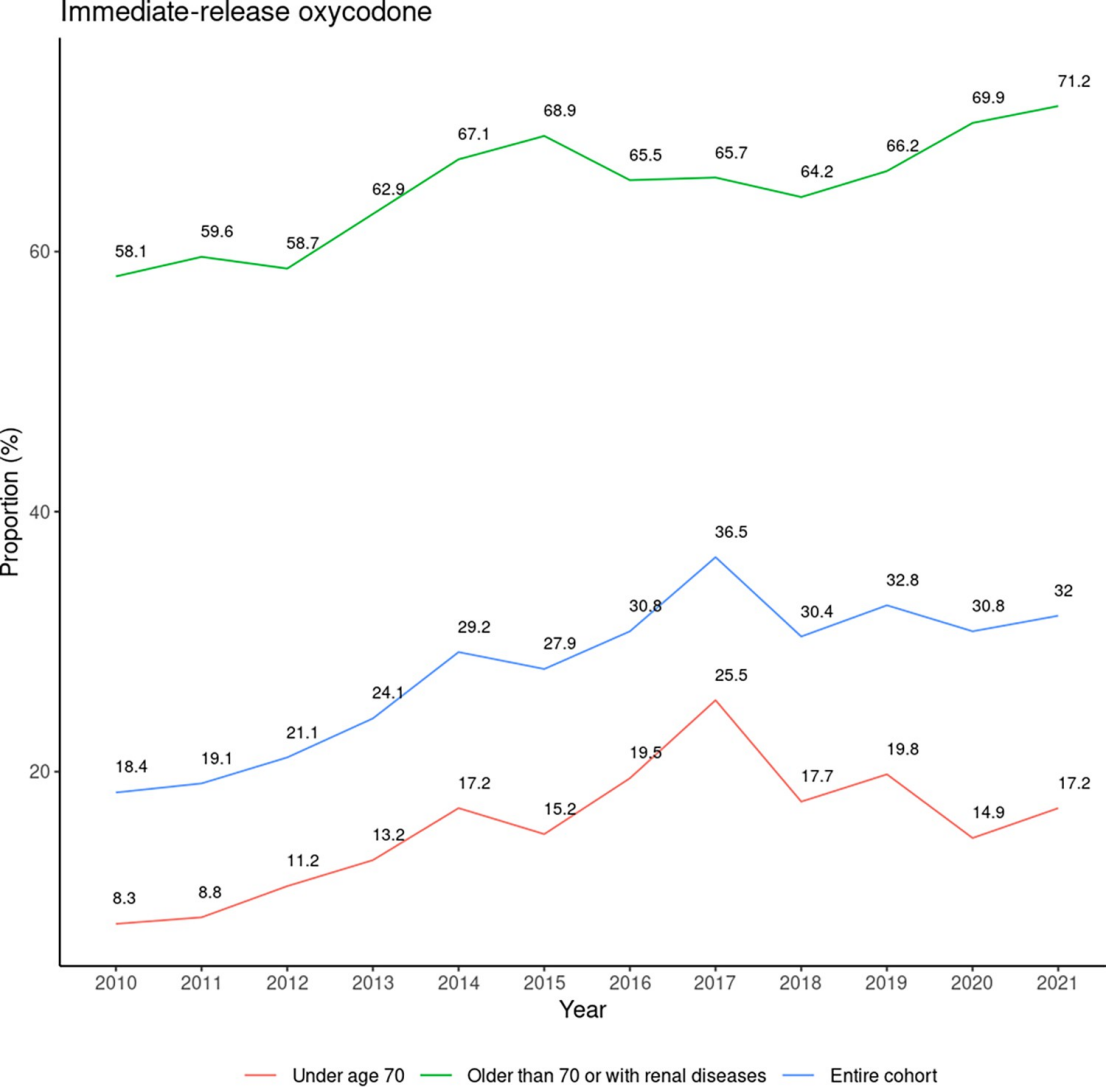
Trends of initial immediate-release oxycodone post-surgery between 2010–2021. The proportions were calculated by dividing the number of patients who took immediate-release oxycodone within 48 hours post-surgery by the number of patients who took any type of opioids within 48 hours post-surgery. Three lines represented a subset of inpatients aged ≤70 years, a subset of inpatients aged>70 years or with renal impairment, and the entire cohort.

**Fig 4:**
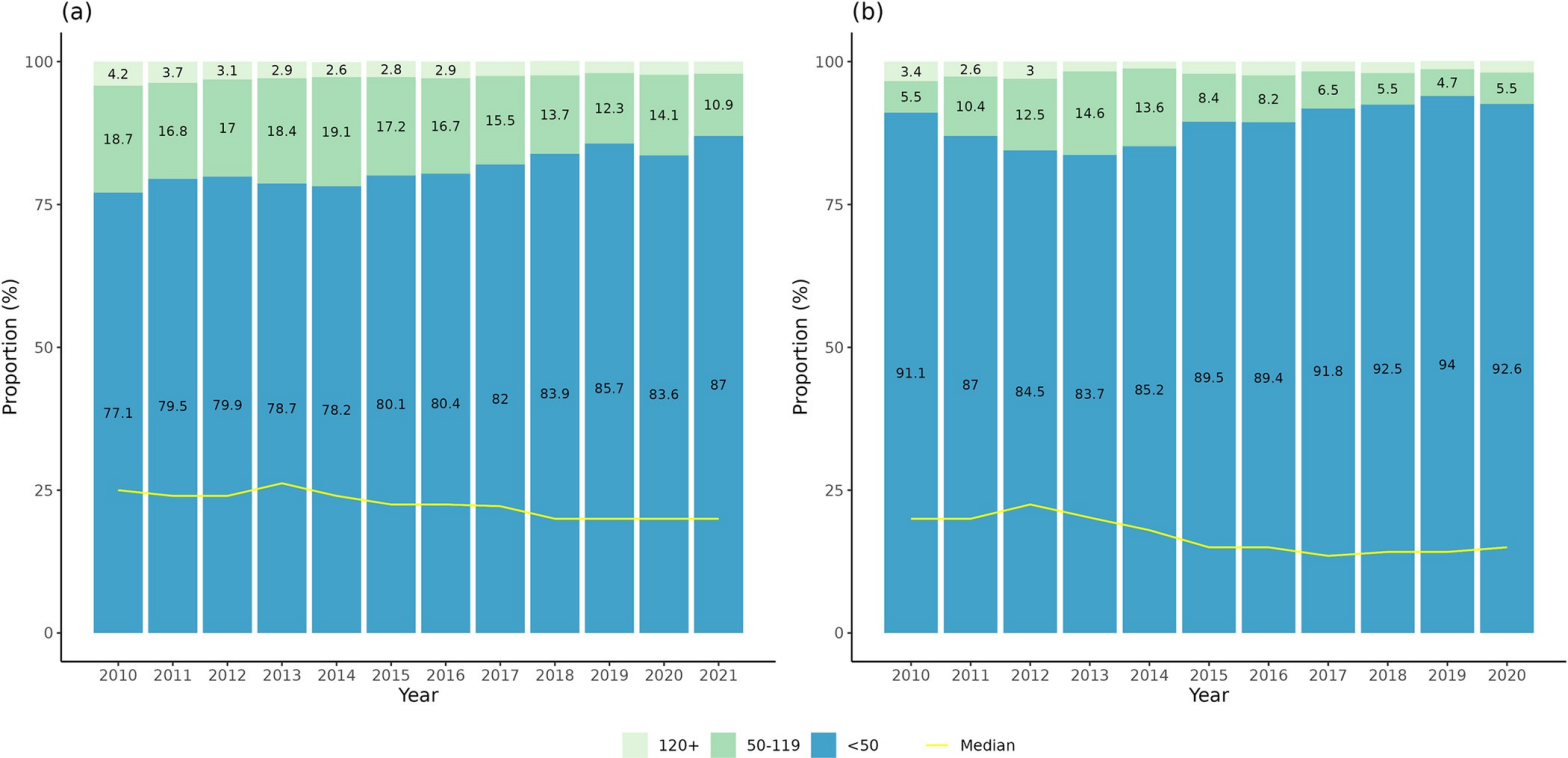
Trends of initial MME/day post-surgery for (a) all inpatients and (b) those aged >70 years or with renal impairment between 2010–2021. In 2021, the median value of MME/day was 11.2. There were 96.1% with MME/day less than 50 and the rest showing MME/day 50 or more. We have not presented data for 2021 due to small-count concerns that might be disclosive of patient information. There were 46,838 admissions for all inpatients and 11,467 admissions for inpatients aged >70 years/ with renal impairment, with opioids administered in the first 48 hours following surgery and their dosage being convertible to MME.

The proportion of MR opioids (e.g., morphine slow release (MST) tablets and fentanyl patches) postoperatively showed an increasing trend from 8.7% to 16% between 2010–2017, dropping initially to 14.0% in 2020 and 11.6% in 2021 (Fig 2). We presented fentanyl patches separately, with a fluctuating percentage of around 2% since 2010 and dropping to 1.3% in 2021. The trend of initial opioid administration of oxycodone IR increased from 18.4% in 2010 to a peak of 36.5% in 2017 (Fig 3). It subsequently decreased and plateaued until 2021 (32.0%). Younger patients (≤70 years) showed a similar trend as the entire cohort, rising from 8.3% to 25.5% between 2010–2017 and dropping to 17.2% in 2021. Instead, those aged >70 years or with renal impairment were observed to have a steady increase from 58.1% to 71.2% (2010–2021). The proportion of all patients on ≥50MME/day dropped from 22.9% (i.e. 18.7%+4.2%) to 13% (i.e. 10.9%+2.1%) between 2010–2021 (Fig 4A). The median value of initial MME/day also reduced from 25.0 to 20.0. Among 60,167 hospital admissions in 50,496 patients who had an opioid administered between 2010–2021, 30,478 admissions (50.7%) in 26,920 patients (53.3%) had an opioid prescribed at discharge following major or orthopaedic surgery.

### Initial opioid use in patients >70 or those with renal impairment

The subgroup comprised 11,482 patients (22.7% of all patients) who contributed to 13,220 unique hospital admissions (22.0%). The most frequent surgical procedure was prosthetic replacement of head of femur, followed by total prosthetic replacement of hip joint and drainage of subdural space in this population (S2 Table). The median value of initial MME/day decreased from 20.0 to 11.2 MME/day between 2010–2021 (Fig 4B). The proportion of patients on ≥50MME/day ranged from 8.9% to 3.9%. Oxycodone was the most common initially administered opioid, with 10.4% administered MR forms and 64.9% IR (S3 Fig). Codeine (29.6% IR), fentanyl (21.2% IR + 1.6% MR = 22.8% in total) and morphine (7.7% IR + 2.0% MR = 9.7% in total) were also commonly used within 48 hours.

## Discussion

This study, to our knowledge, is the first UK study to assess opioid utilisation patterns in the immediate postoperative period using inpatient administered drug data. Amongst patients who underwent major or orthopaedic surgery, 71.1% of patients were administered opioids during their hospitalisation. Some surgery procedures such as laparoscopic procedures may have small wounds, so post-operative opioid therapy may not be necessary and non-opioid alternatives such as paracetamol and non-steroidal anti-inflammatory drugs (NSAIDs) would be sufficient for pain relief. The utilisation of MR opioids doubled from 2010 to 2017 and then slightly dropped, with 1 in 10 taking MR opioids in 2021. Fentanyl patches were administered at a low proportion (~2%) over 10 years and further dropped to 1.3% in 2021 after MHRA’s announcement [8]. Initial use of oxycodone IR more than doubled between 2010–2021 for younger patients (≤70 years), with 1 in 6 administered oxycodone IR within 48 hours following surgery in 2021. We also found a reduction in the proportion of patients on ≥50MME/day for all patients (22.9% to 13%) and the subgroup (8.9% to 3.9%). Oxycodone was frequently administered to inpatients for postsurgical pain management and, for the subgroup, was the most common initially administered opioid that was given to more than two-thirds of patients. Of the patients administered an opioid in hospital, 53.3% were discharged on an opioid.

The high frequency observed in the use of MR opioids and initial oxycodone IR in 2021 is not advocated by the national RCoA guideline released in the same year [4]. The guideline strongly advises prudent care to be exercised when giving MR opioids and avoidance of oxycodone IR as first-line [4,5]. We observed the local hospital guideline for opioid analgesia in acute pain management (developed in 2015 for clinical staff), however, recommends oxycodone to manage moderate to severe acute pain for the subgroup [26] and may explain some of the observations in this high-risk subgroup. Taking the two guidelines altogether, 1 in 6 younger patients (≤70 years) receiving initial oxycodone IR is concerning in practice.

Oral oxycodone administered to the subgroup has better bioavailability and shows a stable plasma level [27], leading to a different recommendation on oxycodone post-surgery between the subgroup [26] and the national RCoA guideline [4]. This may also account for the predominant role of oxycodone in the immediate postoperative period in the subgroup. Oxycodone was also commonly administered to the whole cohort, not only restricted to the subgroup. This is different from primary care prescribing for non-cancer chronic pain in the UK, in which only 0.1% were commenced on oxycodone and codeine was the most frequent drug used, followed by dihydrocodeine [28]. Codeine has been the most prescribed opioid in UK primary care [24,28,29], which may account for the frequent administrations in the immediate postoperative period. This may be driven by the fact that codeine is a weak opioid and aligns with national guidance. In addition, fewer patients received opioids ≥50MME/day as initial treatments following surgery across the decade are promising since it suggests clinicians and prescribers are increasingly cautious about prescribing high-dose or potent opioids to manage postoperative pain.

Limited evidence based largely in North America examines postoperative opioid prescribing using administrative datasets following discharge rather than data from inpatient administration records. Previous work in the US examined the trend for opioid prescribing at surgical discharge between 2010 and 2020 across three diverse healthcare systems serving different populations, including the Veterans Health Administration, Medicaid and an open academic medical centre [16]. The average MME/day at discharge decreased at Veterans Health Administration (37.5–30.1) and Medicaid (41.6–31.3) but increased at academic medical centre (36.9–41.7). An earlier US study assessed the first opioid prescription for outpatient and inpatient (i.e., at discharge) procedures, and showed a noticeable increase in MME between 1994–2014 for some orthopaedic surgeries. For example, the mean MME increased by 145% and 85% for lumbar laminectomy/laminotomy and total hip arthroplasty, respectively [17]. In a Canadian study on adult patients undergoing surgical procedures (e.g., open colectomy and total hip arthroplasty), index MME, identified as the first prescription seven days after either the hospital discharge date or the date of outpatient surgery, increased from 2013 to 2015/2016 and then declined until 2019 [18]. It also showed the most frequently prescribed opioid in 2013 was oxycodone though this changed to hydromorphone in 2019. Although we cannot compare our results to the previous findings directly given the different postoperative periods and data sources, a decreasing trend for initial MME/day in both the whole cohort and subgroup seems to be in line with partial findings of the American and Canadian studies [16,18]. The MME/day values in the American research however are higher than our findings, aligning with likely prescribing practices at the time, but also the use of mean values (median values in this study). Additionally, an Australian study using inpatient electronic data reported 78.5% were administered MR opioids following total hip or knee arthroplasty between 2018–2021. This percentage was much higher than our findings, with 16.2% as the highest between 2010–2021.

Strengths of our study included that it is the first study to our knowledge in the UK providing a snapshot of opioid utilisation patterns in the immediate postoperative period over 11 years in a large UK hospital. It utilised detailed exposure data relating to opioid medications administered to patients when they were hospitalised for surgery; information which is not available using primary care electronic health records (e.g., Clinical Practice Research Datalink). This is especially important when characterising opioid use which can often be prescribed ‘as required’ and therefore reduces exposure misclassification. The results presented, while descriptive, provide a benchmark for future studies and evaluations following national guidance such as RCoA guidelines. There are however several limitations to be considered when interpreting the findings. This was a single-centre UK study rather than national-level inpatient data, which may limit generalisability of the findings. However, currently most NHS hospitals use different electronic health record systems, limiting the availability of a nationally curated dataset. We had information on the surgery date (rather than the exact time) and therefore defined the initial prescribing window as the first 48 hours after surgery covering the day surgery was performed and the following day. We were not able to access opioid prescribing before hospital admissions due to information governance reasons (data entries entered in free text) to identify preoperative opioid use, which may influence postoperative opioid therapy [30,31]. The opioids administered during the operation were likely to be prescribed on handwritten prescription charts and recorded electronically less consistently. Also, we did not have full information on non-opioid analgesics such as paracetamol and NSAIDs in all post-surgical patients as opioids were one of the criteria for data extraction. Lastly, the specific indication for opioid administration is not collected routinely in clinical care as not a requirement for prescribing in the UK, hence unavailable.

### Implications for clinical practice

Our data suggest the high frequency of MR opioids and initial oxycodone IR administered to patients, which are not supported by the Surgery and Opioids Best Practice Guidelines 2021 (i.e. RCoA guidelines) [4]. Approximately 1 in 10 patients were administered MR opioids in 2021 for postoperative pain relief. In 2021, 1 in 3 patients were given initial oxycodone IR while current guidance does not recommend oxycodone IR to be the first line [4]. If focusing on younger patients (≤70 years), the frequency remains as high as 1 in 6 on initial oxycodone IR in 2021. Despite opioids remaining an important component of postoperative pain control, opioid stewardship is recommended by MHRA in 2020 to mitigate harm through optimising, monitoring, and deprescribing opioids [32]. A multimodal opioid-sparing regimen, involving paracetamol and NSAIDs, is also advocated as a best practice after surgical discharge [33]. In addition, whilst the proportion of patients on ≥50MME/day in 2010 was high (>1 in 5 patients), our findings suggest prescribers may be becoming increasingly aware of opioid-related harms, given the decreasing trends of initial MME/day in the whole cohort. The consistently low proportion of fentanyl patches may also attest to the cautiousness of opioids prescribed postoperatively. It would be worth investigating in the future if the level of fentanyl patches keeps decreasing after MHRA’s announcement in 2020 [8], and if they are only prescribed in patients who have tolerated previous opioid therapy.

## Conclusion

In 2021, 1 in 10 patients on MR opioids and 1 in 6 patients ≤70 years were administered initial oxycodone IR following major or orthopaedic surgery. The findings highlight several opportunities to improve safe inpatient prescribing of opioids in line with the national guidance. In 2021, 1 in 8 patients were on high doses of opioids (≥50MME/day) in the initial postoperative period. The decreasing trend of initial MME/day is likely an effort of the increasing awareness and clinical vigilance made by health professionals. There is a pressing need to explore why preference for high-potency opioids remains high, assess their associations with adverse outcomes following surgery, and evaluate whether opioid prescribing practice changes or is improved after the national guideline release.

## Supporting information

S1 Fig Flowchart. Note: In the subsequent calculation of initial MME/day, we further excluded the use of only patient-controlled analgesia (PCA) and injections for recovery only and those without any opioid administered in the first 48 hours, resulting in 46,838 admissions amongst 40,186 patients. There were 11,467 admissions for inpatients aged >70 years/ with renal impairment.
(PDF)

S2 Fig Opioid regimens within one-week post-surgery: Less common combinations (up to 3%). A total of 59,062 admissions with opioids administered in the first seven days between 2010–2021 were included.
(PDF)

S3 Fig Initial opioid types post-surgery for inpatients aged >70 years or with renal impairment. A total of 13,220 admissions with opioids administered to this subgroup in the first 48 hours following surgery between 2010–2021 were included.
(PDF)

S1 Table Most frequent (top 20) surgical procedures. (PDF)

S2 Table Most frequent (top 10) surgical procedures among inpatients aged >70 years or with renal impairment. (PDF)
