# Supplementary material for: Postoperative opioids administered to inpatients with major or orthopaedic surgery: A retrospective cohort study using data from hospital electronic prescribing systems
Source: PLoS One. 2024 Jun 25;19(6):e0305531. doi: 10.1371/journal.pone.0305531 (PMC11198745; doi:10.1371/journal.pone.0305531)
Supplement: S1 Fig — Note: In the subsequent calculation of initial MME/day, we further excluded the use of only patient-controlled analgesia (PCA) and injections for recovery only and those without any opioid administered in the first 48 hours, resulting in 46,838 admissions amongst 40,186 patients. There were 11,467 admissions for inpatients aged >70 years/ with renal impairment. (PDF) [file pone.0305531.s001.pdf]

Figure S1. Flowchart

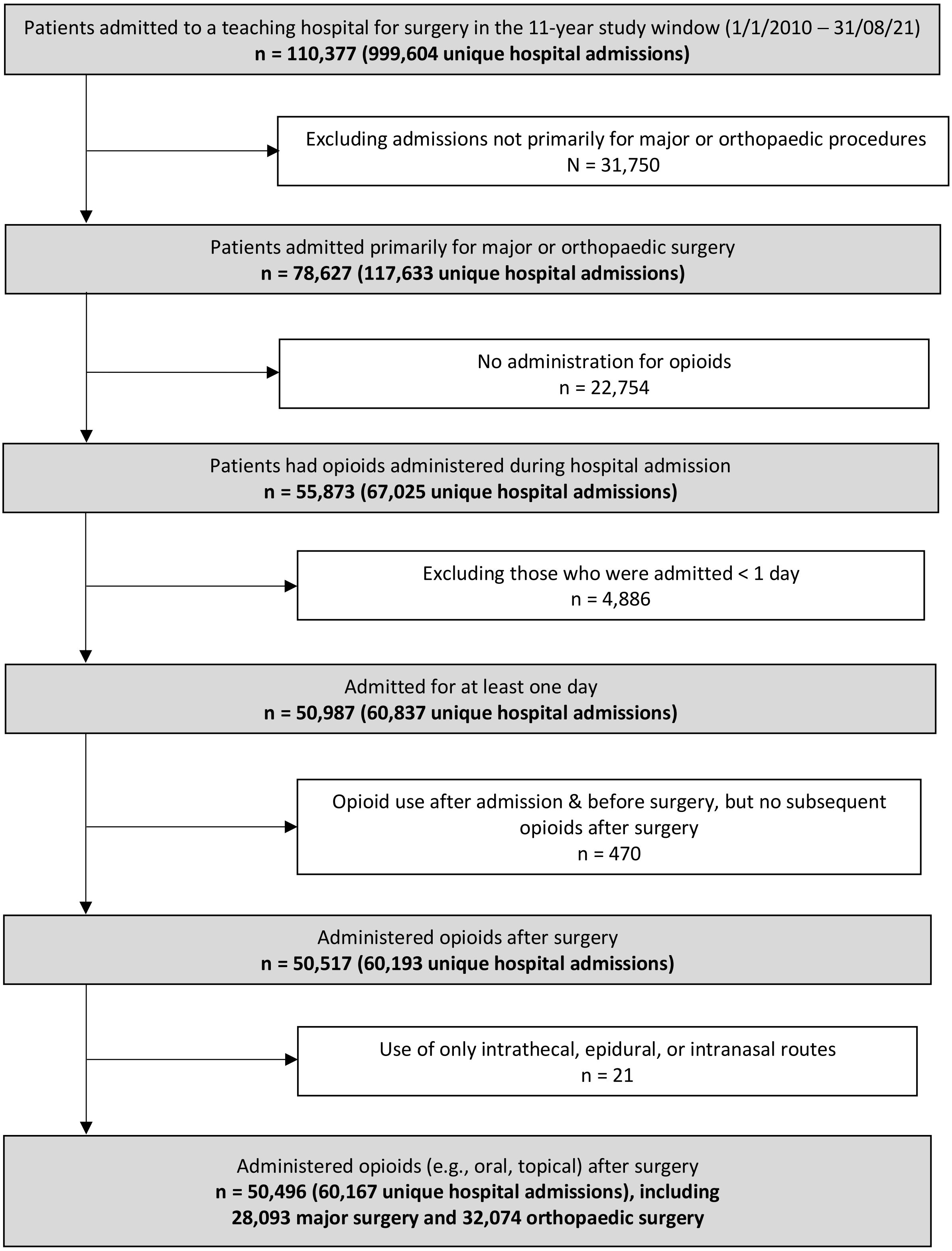

Note: In the subsequent calculation of initial MME/day, we further excluded the use of only patient-controlled analgesia (PCA) and injections for recovery only and those without any opioid administered in the first 48 hours, resulting in 46,838 admissions amongst 40,186 patients. There were 11,467 admissions for inpatients aged >70 years/ with renal impairment.
