# Supplementary material for: Postoperative opioids administered to inpatients with major or orthopaedic surgery: A retrospective cohort study using data from hospital electronic prescribing systems
Source: PLoS One. 2024 Jun 25;19(6):e0305531. doi: 10.1371/journal.pone.0305531 (PMC11198745; doi:10.1371/journal.pone.0305531)
Supplement: S2 Fig — A total of 59,062 admissions with opioids administered in the first seven days between 2010–2021 were included. (PDF) [file pone.0305531.s002.pdf]

Figure S2. Opioid regimens within one-week post-surgery: Less common combinations (up to 3%)

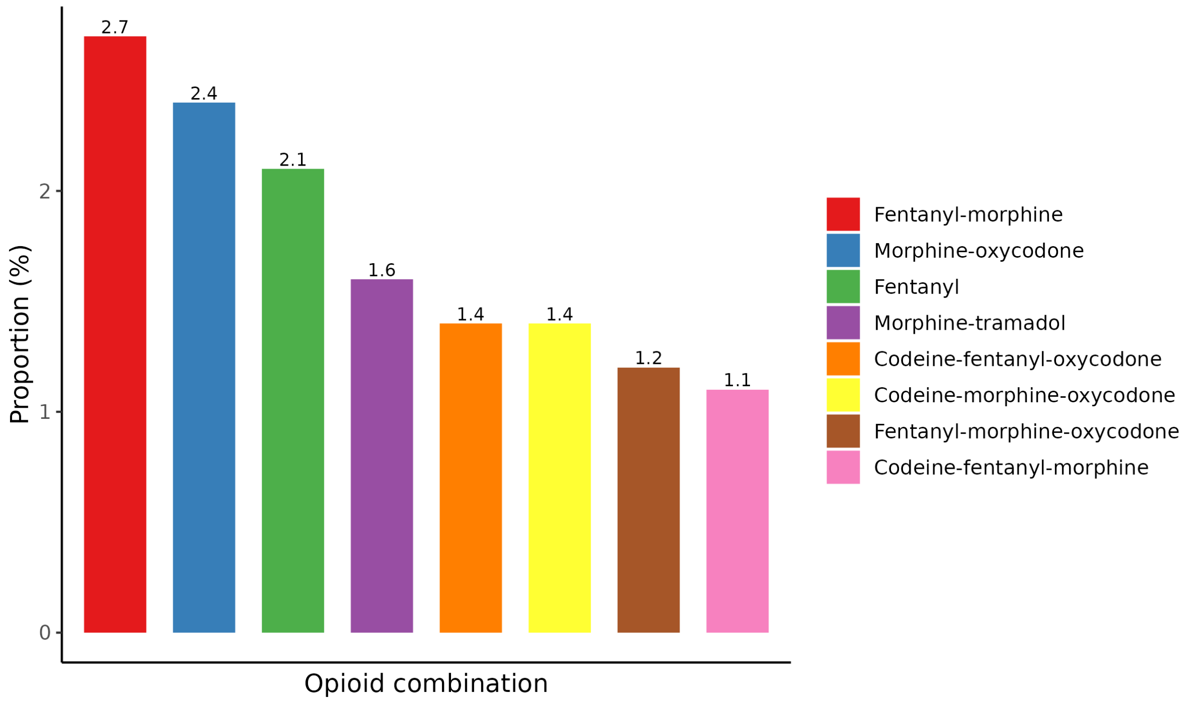

A total of 59,062 admissions with opioids administered in the first seven days between 2010-2021 were included.
