# Supplementary material for: Postoperative opioids administered to inpatients with major or orthopaedic surgery: A retrospective cohort study using data from hospital electronic prescribing systems
Source: PLoS One. 2024 Jun 25;19(6):e0305531. doi: 10.1371/journal.pone.0305531 (PMC11198745; doi:10.1371/journal.pone.0305531)
Supplement: S3 Fig — A total of 13,220 admissions with opioids administered to this subgroup in the first 48 hours following surgery between 2010–2021 were included. (PDF) [file pone.0305531.s003.pdf]

Figure S3. Initial opioid types post-surgery for inpatients aged >70 years or with renal impairment.

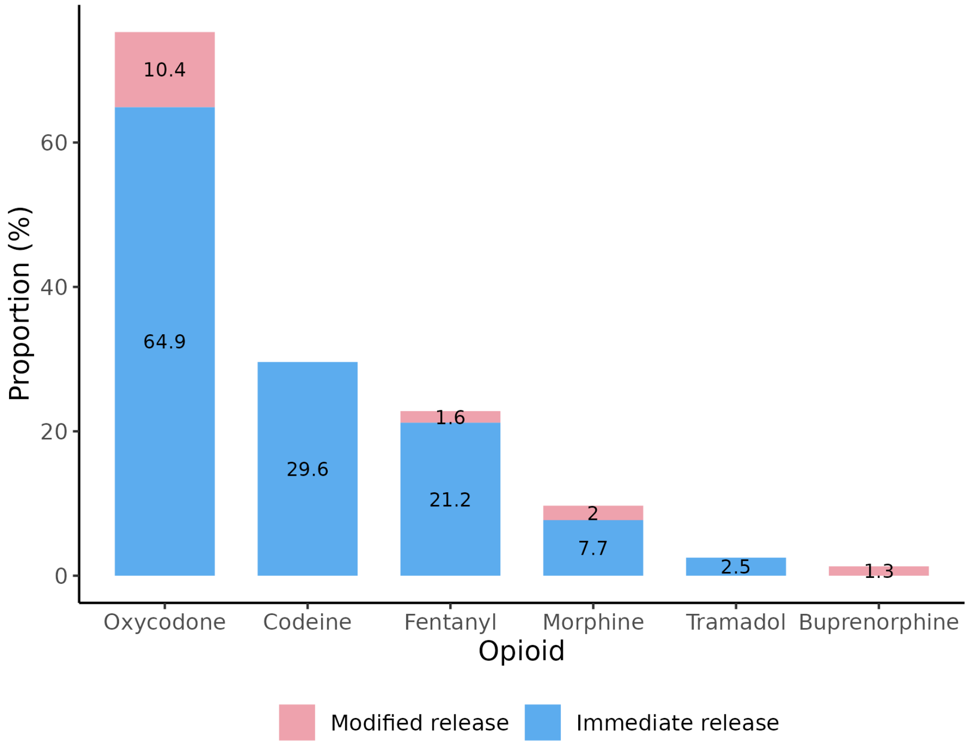

A total of 13,220 admissions with opioids administered to this subgroup in the first 48 hours following surgery between 2010-2021 were included.
