# Supplementary material for: Postoperative opioids administered to inpatients with major or orthopaedic surgery: A retrospective cohort study using data from hospital electronic prescribing systems
Source: PLoS One. 2024 Jun 25;19(6):e0305531. doi: 10.1371/journal.pone.0305531 (PMC11198745; doi:10.1371/journal.pone.0305531)
Supplement: S1 Table — (PDF) [file pone.0305531.s004.pdf]

Table S1. Most frequent (top 20) surgical procedures

Surgery type	Surgery description	Type	N	%
Primary decompression operations on lumbar spine	Other specified	Orthopaedic	2618	4.4%
Primary excision of cervical intervertebral disc	Primary anterior excision of cervical intervertebral disc an	Orthopaedic	1805	3.0%
Excision of gall bladder	Total cholecystectomy nec	Major	1701	2.8%
Primary decompression operations on lumbar spine	Primary posterior decompression of lumbar spine nec	Orthopaedic	1530	2.5%
Drainage of subdural space	Evacuation of subdural haematoma	Major	1214	2.0%
Instrumental correction of deformity of spine	Posterior attachment of correctional instrument to spine	Orthopaedic	999	1.7%
Prosthetic replacement of head of femur using cement	Primary prosthetic replacement of head of femur using cement	Orthopaedic	976	1.6%
Primary excision of lumbar intervertebral disc	Primary microdiscectomy of lumbar intervertebral disc	Orthopaedic	936	1.6%
Total prosthetic replacement of knee joint using cement	Primary total prosthetic replacement of knee joint using cement	Orthopaedic	854	1.4%
Total prosthetic replacement of hip joint using cement	Primary total prosthetic replacement of hip joint using cement	Orthopaedic	820	1.4%
Puncture of joint	Aspiration of joint	Orthopaedic	810	1.3%
Primary open reduction of fracture of bone and extramed	Primary open reduct/fracture/long bone/extramed/fix using pl	Orthopaedic	804	1.3%
Primary decompression operations on lumbar spine	Primary posterior laminectomy decompression of lumbar spine	Orthopaedic	797	1.3%
Abdominal excision of uterus	Total abdominal hysterectomy nec	Major	764	1.3%
Operations on tissue of brain	Monitoring of pressure in tissue of brain	Major	692	1.2%
Excision of lesion of tissue of brain	Excision of lesion of tissue of frontal lobe of brain	Major	652	1.1%
Primary decompression operations on cervical spine	Primary anterior decompression of cervical spinal cord and f	Orthopaedic	630	1.0%
Creation of connection from ventricle of brain	Creation of ventriculoperitoneal shunt	Major	574	1.0%
Other operations on pituitary gland	Excision of lesion of pituitary gland	Major	564	0.9%
Emergency excision of appendix	Emergency excision of abnormal appendix nec	Major	563	0.9%
