# Supplementary material for: Postoperative opioids administered to inpatients with major or orthopaedic surgery: A retrospective cohort study using data from hospital electronic prescribing systems
Source: PLoS One. 2024 Jun 25;19(6):e0305531. doi: 10.1371/journal.pone.0305531 (PMC11198745; doi:10.1371/journal.pone.0305531)
Supplement: S2 Table — (PDF) [file pone.0305531.s005.pdf]

Table S2. Most frequent (top 10) surgical procedures among inpatients aged >70 years or with renal impairment

Surgery type	Surgery description	Type	N	%
Prosthetic replacement of head of femur using cement	Primary prosthetic replacement of head of femur using cement	Orthopaedic	815	6.2%
Total prosthetic replacement of hip joint using cement	Primary total prosthetic replacement of hip joint using ceme	Orthopaedic	512	3.9%
Drainage of subdural space	Evacuation of subdural haematoma	Major	454	3.4%
Total prosthetic replacement of knee joint using cement	Primary total prosthetic replacement of knee joint using cem	Orthopaedic	398	3.0%
Puncture of joint	Aspiration of joint	Orthopaedic	354	2.7%
Primary decompression operations on lumbar spine	Primary posterior decompression of lumbar spine nec	Orthopaedic	347	2.6%
Closed reduction of fracture of bone and internal fixat	Closed reduction fracture bone fixation using nail or screw	Orthopaedic	331	2.5%
Primary decompression operations on lumbar spine	Other specified	Orthopaedic	330	2.5%
Closed reduction of fracture of bone and internal fixat	Closed reduction of intracapsular fracture of neck of femur	Orthopaedic	315	2.4%
Primary decompression operations on lumbar spine	Primary posterior laminectomy decompression of lumbar spine	Orthopaedic	279	2.1%
